# Comparative effectiveness of empagliflozin versus dapagliflozin in adults with metabolic dysfunction-associated steatotic liver disease

**DOI:** 10.3389/fendo.2025.1669613

**Published:** 2025-10-13

**Authors:** Jheng-Yan Wu, Yu-Kuan Tu, Chia-Chih Kuo, Mei-Yuan Liu, Wan-Hsuan Hsu, Ya-Wen Tsai, Ting-Hui Liu, Po-Yu Huang, Min-Hsiang Chuang, Kuo-Chuan Hung, Tsung Yu, Kuang-Ming Liao, Chih-Cheng Lai

**Affiliations:** ^1^ Department of Nutrition, Chi Mei Medical Center, Tainan, Taiwan; ^2^ Department of Public Health, College of Medicine, National Cheng Kung University, Tainan, Taiwan; ^3^ Department of Internal Medicine, Chi Mei Medical Center, Tainan, Taiwan; ^4^ Department of Nutrition and Health Sciences, Chang Jung Christian University, Tainan, Taiwan; ^5^ Department of Food Nutrition, Chung Hwa University of Medical Technology, Tainan, Taiwan; ^6^ Department of Health and Nutrition, Chia Nan University of Pharmacy & Science, Tainan, Taiwan; ^7^ Division of Preventive Medicine, Chi Mei Medical Center, Tainan, Taiwan; ^8^ Department of Psychiatry, Chi Mei Medical Center, Tainan, Taiwan; ^9^ Division of Nephrology, Department of Internal Medicine, Chi Mei Medical Center, Tainan, Taiwan; ^10^ Department of Anesthesiology, Chi Mei Medical Center, Tainan, Taiwan; ^11^ Department of Internal Medicine, Chi Mei Medical Center, Chiali, Taiwan; ^12^ Department of Nursing, Min-Hwei Junior College of Health Care Management, Tainan, Taiwan; ^13^ Department of Intensive Care Medicine, Chi Mei Medical Center, Tainan, Taiwan; ^14^ School of Medicine, College of Medicine, National Sun Yat-sen University, Kaohsiung, Taiwan

**Keywords:** empagliflozin, dapagliflozin, diabetes mellitus, metabolic dysfunction-associated steatotic liver disease, sodium-glucose co-transporter-2 inhibitor

## Abstract

**Background:**

Sodium-glucose co-transporter-2 inhibitors (SGLT2is) show promise in treating metabolic dysfunction-associated steatotic liver disease (MASLD). However, the relative efficacy of different SGLT2is remains unclear. We aimed to compare the clinical effectiveness of empagliflozin versus dapagliflozin in adults with MASLD.

**Methods:**

Using the TriNetX database, we conducted a retrospective cohort study of adults with MASLD who were newly prescribed either empagliflozin or dapagliflozin between January 2013 and September 2024. After propensity score matching, we compared 13,274 patients in each group. The primary outcome was a composite of all-cause hospitalization, all-cause mortality, major adverse cardiovascular events (MACEs), major adverse kidney events (MAKEs), and decompensated hepatic events. Secondary outcomes included each individual component of the primary outcome.

**Results:**

Empagliflozin was associated with a lower risk of primary composite outcomes compared to dapagliflozin (HR, 0.84; 95% CI, 0.80-0.88). This benefit was consistent across most subgroups, including sex, presence of liver cirrhosis, heart failure, T2DM, and chronic kidney disease. Significant interactions were observed for age groups (p=0.04) and borderline for BMI categories (p=0.06). Empagliflozin also showed lower risks for all-cause hospitalization (HR, 0.84; 95% CI, 0.79-0.88), all-cause mortality (HR, 0.79; 95% CI, 0.66-0.96), MACE (HR, 0.88; 95% CI, 0.78-0.99), and MAKE (HR, 0.63; 95% CI, 0.47-0.86), but no difference in decompensated hepatic events (HR, 1.01; 95% CI, 0.81-1.27).

**Conclusions:**

In patients with MASLD, empagliflozin was associated with better clinical outcomes compared to dapagliflozin, particularly in reducing cardiovascular and renal events, hospitalizations, and mortality.

## Introduction

Metabolic dysfunction-associated steatotic liver disease (MASLD), formerly known as nonalcoholic fatty liver disease (NAFLD), has emerged as the most prevalent liver condition worldwide, affecting an estimated 38% of the global adult population (1–3). MASLD is a complex, multisystem disorder with implications extending far beyond hepatic health, such as liver cirrhosis and hepatocellular carcinoma (HCC). Its impact encompasses various extra-hepatic complications, including cardiovascular disease, extra-hepatic cancers, type 2 diabetes mellitus (T2DM), and chronic kidney disease (CKD). These associated conditions, along with liver-related issues, significantly contribute to increased morbidity and mortality rates among MASLD patients (4–6). The diverse range of potential complications underscores the need for a comprehensive, multidisciplinary approach to MASLD management. This holistic strategy must address both the hepatic and extra-hepatic manifestations of the disease to ensure optimal patient outcomes (5, 7).

Therapeutic strategies for MASLD remain limited due to insufficient evidence supporting their beneficial effects. In this context, sodium-glucose co-transporter-2 inhibitors (SGLT2is) with pleiotropic effects have emerged as a promising option. SGLT2is were originally approved for treating T2DM, but some, such as empagliflozin and dapagliflozin, have demonstrated beneficial effects on cardiovascular and renal outcomes, leading to their additional approval for CKD and heart failure management (8). Building on these established indications, recent investigations have begun exploring the potential of SGLT2is in treating MASLD, offering hope for a more effective multisystem approach to this complex disorder (7, 9).

Several retrospective and population-based studies (10–13) have shown promising results for SGLT2is in managing MASLD. Two retrospective nationwide claims database studies (10, 11) in Japan demonstrated that SGLT2is, compared to dipeptidyl peptidase-4 inhibitors (DPP4i), were associated with greater improvement in fatty liver index, hepatic inflammation and fibrosis indices, and a reduced incidence of esophageal varices in patients with MASLD and T2DM. A population-based cohort study in Korea revealed that SGLT2i was associated with a lower risk of hepatic decompensation events in patients with MASLD compared to thiazolidinediones (TZD) (12). Similarly, a US-based cohort study using the Merative Marketscan Research Databases showed that SGLT2i was associated with significantly lower risks of HCC, liver cirrhosis, cardiovascular disease (CVD), CKD, and extra-hepatic cancer compared to other anti-diabetic medications (13). While these findings indicate the potential role of SGLT2i in managing patients with MASLD and T2DM, it remains unclear whether all SGLT2is share similar benefits in this specific population. Therefore, this study aims to compare the clinical effectiveness of empagliflozin versus dapagliflozin in adults with MASLD.

## Methods

### Data source and cohort

This cohort study used data from the TriNetX database, which collects deidentified, patient-level data from electronic health records. Information in the TriNetX database comes from health care organizations (HCOs), typically academic health care centers, that collect data from their main and satellite hospitals and outpatient clinics. Available data include demographics, diagnoses (based on International Classification of Diseases, Tenth Revision, Clinical Modification codes), procedures (classified by International Classification of Diseases, Tenth Revision Procedure Coding System or Current Procedural Terminology), medications (Veterans Affairs Drug Classification System and RxNorm codes), laboratory tests (organized using Logical Observation Identifiers Names and Codes), and health care utilization records. We used the Global Collaborative Network in TriNetX, which includes data from more than 155 million patients from 32 HCOs in the database.

The analysis of TriNetX data in this cohort study was approved by the institutional review board of Chi Mei Hospital, Tainan, Taiwan (Chi Mei Hospital’s institutional review board approval number: 11402-E02). Additionally, the TriNetX platform maintains compliance with the Health Insurance Portability and Accountability Act and General Data Protection Regulation, ensuring the utmost protection of patient information. The platform aggregates and consolidates only counts and statistical summaries of deidentified data from various institutions, without containing any individual-level data. Consequently, the Western Institutional Review Board has granted a waiver of informed consent for use of TriNetX data due to the absence of individual-level information. The current study was followed the Strengthening the Reporting of Observational Studies in Epidemiology (STROBE) reporting guideline.

### Study population and exposure

We included patients aged 18 years or older diagnosed with MASLD who were prescribed either empagliflozin or dapagliflozin between January 1, 2013, and September 30, 2024, following the US Food and Drug Administration’s 2013 approval of SGLT2i. Patients with any prespecified outcomes occurring before the index date were excluded to ensure accurate identification of incident outcomes. The patients were divided into empagliflozin and dapagliflozin groups, excluding those with any prior prescriptions of the counterpart medication (e.g., patients in the empagliflozin group with prior dapagliflozin use) within 6 months before or at any time after the index date. An incident user design was achieved by excluding patients with any prior use of empagliflozin or dapagliflozin before the index date. Additionally, to prevent potential misclassification, patients with stage 5 CKD or end-stage kidney disease (ESKD) at baseline were excluded. Details regarding the codes used to identify the demographics, diagnoses, procedures, medications, and laboratory results are provided in **eTable 1**.

### Covariates

From each patient, we extracted covariables from study days −365 to 0 for inclusion in propensity score models that we thought were likely to confound the association between SGLT2i selection and the prespecified outcomes. Covariables included demographics, cardiac and metabolic-related diagnoses and medication use, glomerular filtration rate, and hemoglobin A1c. A complete list of covariables and covariable definitions are included in **eTable 2**.

### Outcomes

The primary outcome was a composite of all-cause hospitalization, all-cause mortality, major adverse cardiovascular events (MACEs), major adverse kidney events (MAKEs), and decompensated hepatic events occurring during the follow-up period. Secondary outcomes included each individual component of the primary outcome. MACEs were defined as myocardial infarction, ischemic or hemorrhagic stroke, or cardiac death, while MAKEs were defined as progression to stage 5 CKD, ESKD, or initiation of dialysis. Additionally, sensorineural hearing loss, skin cancer, and lumbar radiculopathy as negative control outcome were included as a negative control exposure. Diagnostic, visit, and procedural codes used to identify these outcomes are provided in **eTable 3**.

### Subgroup and sensitivity analysis

We conducted prespecified subgroup analyses stratified by sex, age (18–64 years vs. ≥65 years), baseline BMI (<30 vs. ≥30 kg/m²), and the presence of conditions including nonalcoholic steatohepatitis (NASH), liver cirrhosis, heart failure, T2DM, and CKD.

### Statistical analysis

Baseline characteristics for participants in the empagliflozin and dapagliflozin groups were presented based on data type, with categorical variables displayed as counts and percentages, and continuous variables as means with standard deviations. To balance these baseline characteristics across groups, one-to-one PSM was implemented using a greedy nearest neighbor approach with a 0.1 pooled standard deviation caliper. Variables were considered balanced if the standardized difference between groups was under 0.1 (14). Survival probabilities were estimated via the Kaplan-Meier method after matching.

Hazard ratios (HRs) with 95% confidence intervals (CIs) and associated P-values were computed using Cox proportional hazards regression for all outcome measures. Unmeasured confounding was assessed through the E-value approach, estimating the minimum strength of association required between an unmeasured confounder and both the exposure and outcome to explain observed differences. For instance, an E-value of x signifies that an unmeasured confounder would need an association of at least x-fold with both exposure and outcome to negate the observed association after accounting for measured confounders (15).

Statistical significance was assessed with two-sided tests, setting P <.05. To control for multiple comparisons across six primary and secondary outcomes, a Bonferroni-adjusted alpha level of.0083 was applied, ensuring an overall type I error rate of.0488. Subgroup interaction was examined by evaluating confidence interval overlap, assessing the significance of observed differences (16). All analyses were conducted using the TriNetX analytical platform.

## Results

### The selection of patients

**Figure 1:**
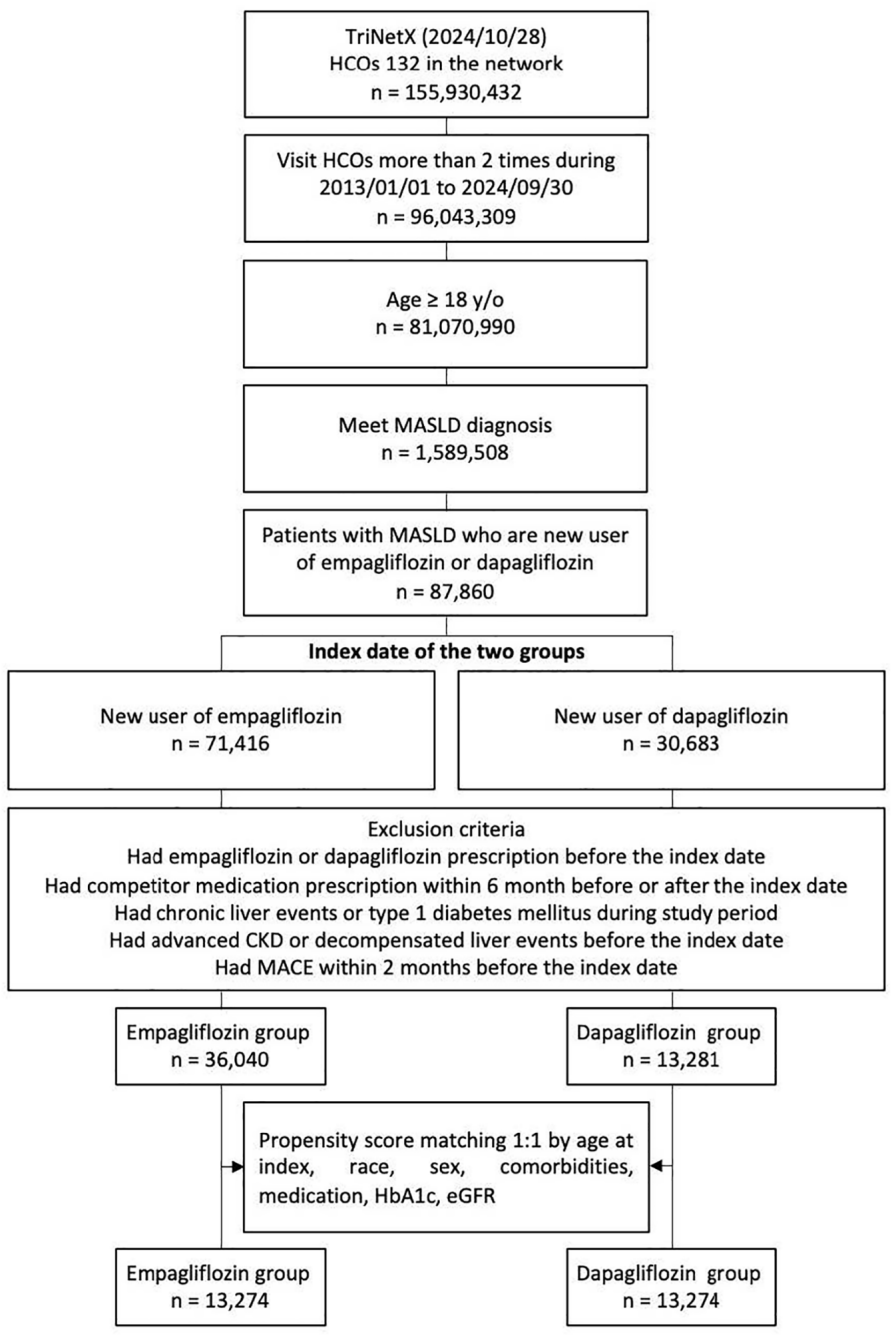
Algorithm of patient selection.

This study analyzed data from the TriNetX network (as of October 28, 2024), which encompasses 132 HCOs and 155,930,432 individuals. We included patients aged 18 or older who had made at least two HCO visits between 2013 and 2024, yielding 81,070,990 eligible individuals. Among these, we identified 1,589,508 patients with MASLD. Of this group, 87,860 patients were new users of either empagliflozin (n = 71,416) or dapagliflozin (n = 30,683). After applying exclusion criteria, the cohort comprised 36,040 empagliflozin users and 13,281 dapagliflozin users. Through 1:1 PSM, we established a final cohort of 13,274 patients in each group. Algorithm of patient selection are summarized in **Figure 1**.

### Demographic features of included patients

**Table 1** shows the comparison between empagliflozin and dapagliflozin groups before and after PSM. Before matching, there were no significant differences in age, distribution of sex, and race between groups. Additionally, no significant differences were observed between groups in the prevalence of comorbidities, except that the dapagliflozin group had a higher prevalence of heart failure and T2DM than the empagliflozin group (18.3% vs. 13.6% and 74.9% vs. 69.3%). Regarding laboratory examinations, the empagliflozin group had better renal function but higher HbA1c levels than the dapagliflozin group. Lastly, there were no significant differences between groups in medications, except that more biguanides were prescribed in the empagliflozin group than in the dapagliflozin group. After PSM, 13,274 matched patients were yielded in each cohort, with standardized differences in all covariates between groups less than 0.1 (**Table 1**).

**Table 1:** Baseline characteristics of study population before and after propensity score matching.

Variables	Before matching			After matching		
	Empagliflozin (n=36 262)	Dapagliflozin (n=12 951)	Standardized difference	Empagliflozin (n=12 951)	Dapagliflozin (n=12 951)	Standardized difference
Age at index, years						
Mean (SD)	59.3 (12.7)	59.4 (13.1)	0.006	59.4 (13.0)	59.4 (13.1)	0.002
Gender, n(%)						
Female	17 703 (48.8)	6 515 (50.3)	0.029	6 593 (50.9)	6 512 (50.3)	0.013
Male	17 140 (47.3)	6 228 (48.0)	0.016	6 165 (47.6)	6 220 (48.0)	0.009
Race, n(%)						
White	24 323 (67.1)	8 810 (68)	0.019	8 828 (68.2)	8 803 (68)	0.004
Black or African American	3 660 (10.1)	1 162 (9.0)	0.038	1 200 (9.3)	1 162 (9.0)	0.010
Asian	1 872 (5.2)	754 (5.8)	0.029	725 (5.6)	753 (5.8)	0.009
Other Race	1 601 (4.4)	593 (4.6)	0.008	586 (4.5)	592 (4.6)	0.002
Unknown Race	4 409 (12.2)	1 533 (11.8)	0.010	1 490 (11.5)	1 531 (11.8)	0.010
Comorbidities, n(%)						
Overweight and obesity	12, 510 (34.5)	4 155 (32.1)	0.052	4 071 (31.4)	4 151 (32.1)	0.013
Alcohol related disorders	743 (2.0)	317 (2.4)	0.027	320 (2.5)	315 (2.4)	0.002
Nicotine dependence	3 106 (8.6)	1 131 (8.7)	0.006	1 122 (8.7)	1 129 (8.7)	0.002
Type 2 diabetes mellitus	27 168 (74.9)	8 975 (69.3)	0.174	8 795 (67.9)	8 973 (69.3)	0.029
Hypertension	23 154 (63.9)	7 896 (60.9)	0.061	7 678 (59.3)	7 891 (60.9)	0.034
Hyperlipidemia	22 465 (62.0)	7 641 (58.9)	0.061	7 452 (57.5)	7 631 (58.9)	0.028
Cerebrovascular diseases	1 764 (4.9)	709 (5.5)	0.027	657 (5.1)	707 (5.5)	0.017
Chronic lower respiratory diseases	6 983 (19.3)	2 557 (19.7)	0.012	2 507 (19.4)	2 552 (19.7)	0.009
Hypertensive diseases	24 176 (66.7)	8 441 (65.1)	0.033	8 228 (63.5)	8 430 (65.1)	0.033
Ischemic heart diseases	7 480 (20.6)	2 973 (22.9)	0.056	2 898 (22.4)	2 965 (22.9)	0.012
Heart failure	4 934 (13.6)	2 372 (18.3)	0.128	2 312 (17.9)	2 361 (18.2)	0.010
Atrial fibrillation and flutter	3 363 (9.3)	1 458 (11.2)	0.065	1 419 (11)	1 452 (11.2)	0.008
Peripheral vascular disease	1 185 (3.3)	503 (3.9)	0.033	478 (3.7)	502 (3.9)	0.010
Neoplasms	8 140 (22.4)	2 724 (21)	0.035	2 753 (21.3)	2 722 (21)	0.006
Systemic connective tissue disorders	755 (2.1)	269 (2.1)	<0.001	255 (2.0)	268 (2.1)	0.007
Fatty liver	14 466 (39.9)	5 091 (39.3)	0.013	4 967 (38.4)	5 082 (39.2)	0.018
Nonalcoholic steatohepatitis	2 412 (6.7)	742 (5.7)	0.038	708 (5.5)	742 (5.7)	0.011
Hepatic fibrosis	501 (1.4)	176 (1.4)	0.002	170 (1.3)	176 (1.4)	0.004
Cirrhosis of liver	1 139 (3.1)	429 (3.3)	0.010	436 (3.4)	429 (3.3)	0.003
Glomerular filtration rate, mL/min/1.73 m^2^						
Mean (SD)	80.0 (26.0)	76.1 (27.2)	0.145	77.1 (27.2)	76.1 (27.2)	0.035
< 45 mL/min/1.73 m^2^	3 998 (11.0)	2 264 (17.5)	0.144	2 202 (17.0)	2 243 (17.3)	0.008
HbA1c, %						
Mean (SD)	8.0 (1.8)	7.8 (1.9)	0.110	7.8 (1.9)	7.8 (1.9)	0.020
≥ 9%	7 422 (20.5)	2 145 (16.6)	0.101	2 125 (16.4)	2 145 (16.6)	0.004
Medications						
HMG CoA reductase inhibitors	17 932 (49.5)	6 003 (46.3)	0.063	5 961 (46.0)	5 993 (46.3)	0.005
Diuretics	12 001 (33.1)	4 492 (34.7)	0.033	4 389 (33.9)	4 482 (34.6)	0.015
Beta blockers	11 446 (31.6)	4 332 (33.4)	0.040	4 272 (33.0)	4 322 (33.4)	0.008
Calcium channel blockers	7 786 (21.5)	2 846 (22.0)	0.012	2 822 (21.8)	2 838 (21.9)	0.003
ACEis/ARBs	17 131 (47.2)	5 934 (45.8)	0.029	5 862 (45.3)	5 926 (45.8)	0.010
Antiarrhythmics	10 011 (27.6)	3 642 (28.1)	0.011	3 518 (27.2)	3 633 (28.1)	0.020
Biguanides	16 246 (44.8)	4 610 (35.6)	0.189	4 620 (35.7)	4 609 (35.6)	0.002
Sulfonylureas	6 018 (16.6)	1 779 (13.7)	0.080	1 777 (13.7)	1 779 (13.7)	0
Thiazolidinediones	1 007 (2.8)	381 (2.9)	0.010	368 (2.8)	381 (2.9)	0.006
Dipeptidyl peptidase 4 inhibitors	2 834 (7.8)	1 025 (7.9)	0.003	1 027 (7.9)	1 025 (7.9)	0.001
Glucose-like peptide-1 analogues	5 194 (21.0)	1 573 (17.2)	0.010	1 581 (17.3)	1 573 (17.2)	0.002
Insulins and analogues	9 631 (26.6)	3 481 (26.9)	0.007	3 424 (26.4)	3 476 (26.8)	0.009

ACEi, angiotensin-converting enzyme inhibitors; ARB, angiotensin receptor blockers; SD, standard deviation.

Standardized difference < 0.1 is considered a small difference.

### Primary outcome

**Figure 2:**
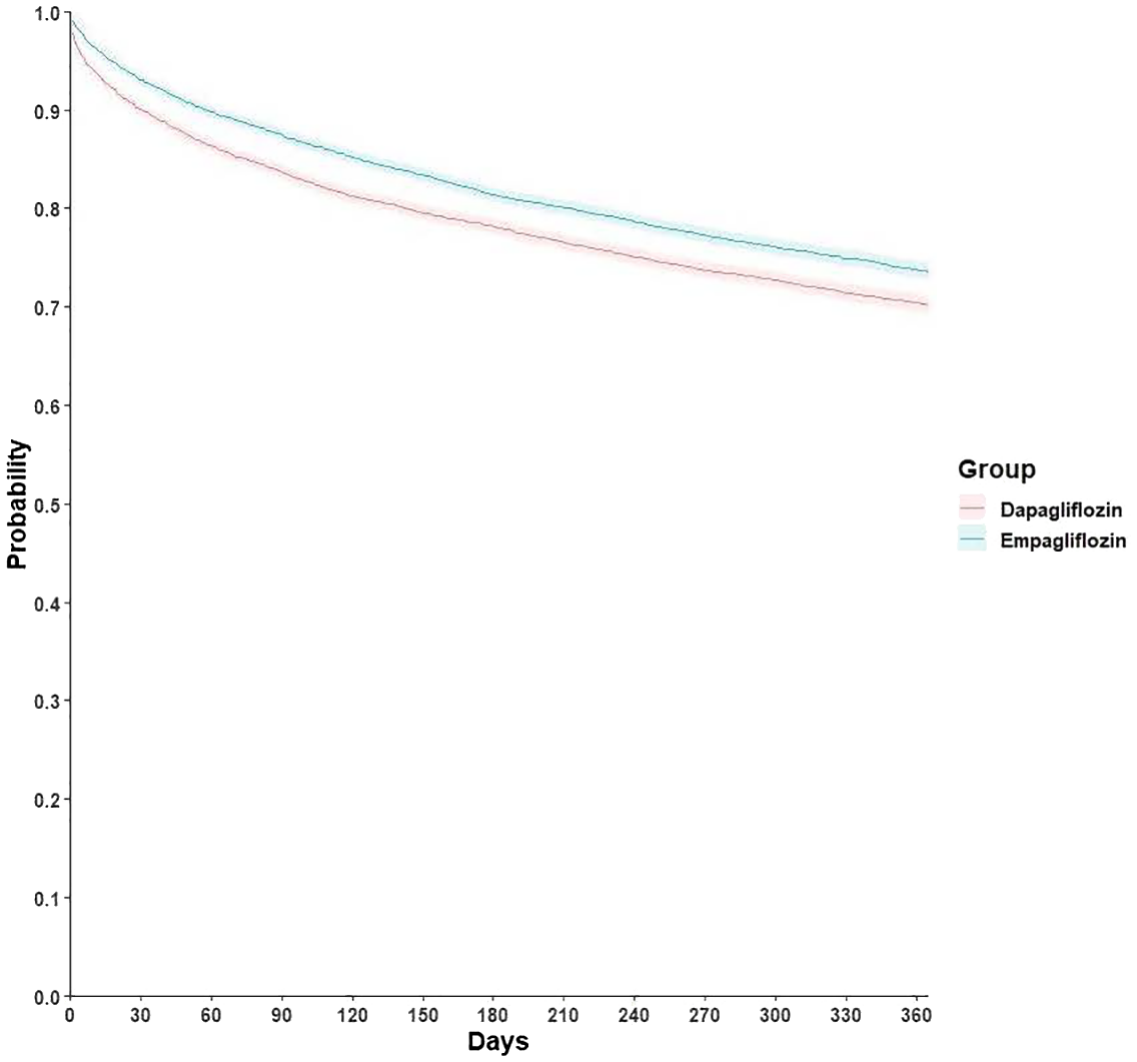
Kaplan-Meier curves of primary composite outcome among empagliflozin and dapagliflozin groups.

During follow-up, the empagliflozin group was associated with a lower risk of primary composite outcomes than the dapagliflozin group (HR, 0.84; 95% CI, 0.80-0.88, **Table 2**). The test for proportionality showed no violation of the proportional hazard assumption (Schoenfeld test p-value > 0.05) and an E-value of 1.51 was observed, indicating strong robustness against potential unmeasured confounders. Survival analysis showed a lower cumulative incidence of primary outcome in the empagliflozin group (log-rank test, p < 0.001) (**Figure 2**).

**Table 2:** Comparison of empagliflozin vs dapagliflozin for primary and secondary outcomes.

Outcome	No. of patients with outcome		HR (95% CI)	*P* value	E-value (e-value for the upper limit of CI)
	Empagliflozin (n = 12 951)	Dapagliflozin (n = 12 951)			
Primary outcome					
Composites of secondary outcomes	2 890	3 363	0.84 (0.80,0.88) ^a^	<.001 ^b^	1.51 (1.41)
Secondary outcomes					
All-cause Hospitalization	2 564	2 988	0.84 (0.79,0.88) ^a^	<.001 ^b^	1.51 (1.41)
All-cause mortality	198	250	0.79 (0.66,0.96) ^a^	0.015 ^b^	2.26 (1.25)
MACEs	491	561	0.88 (0.78,0.99) ^a^	0.032 ^b^	1.53 (1.11)
MAKEs	66	104	0.63 (0.47,0.86) ^a^	0.004 ^b^	2.55 (1.60)
Decompensated hepatic events	150	148	0.99 (0.79,1.25) ^a^	0.957 ^b^	1.11 (1)

CI, confidence interval; HR, hazard ratio; MACE, major adverse cardiovascular event; MAKE, major adverse kidney event.

^a^Proportionality (Schoenfeld test *P*>.05).

^b^Bonferroni-corrected α= .0083.

**Figure 3:**
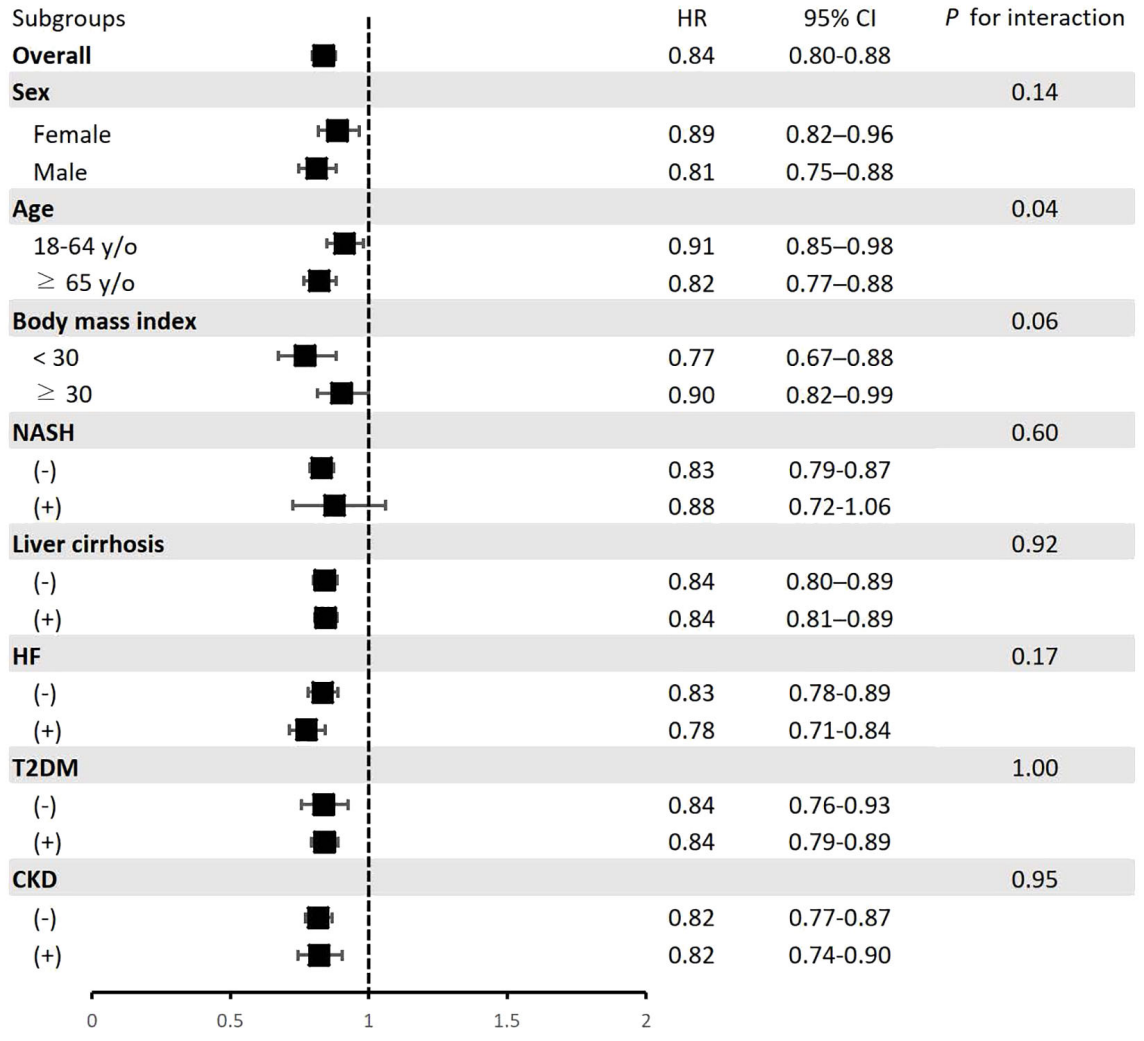
Subgroup analysis of primary outcomes between empagliflozin and dapagliflozin groups.

Further stratified analysis showed that compared to dapagliflozin, empagliflozin was associated with a lower risk of primary outcomes in both males (HR, 0.81; 95% CI, 0.75-0.88) and females (HR, 0.89; 95% CI, 0.82-0.96), as well as across age groups with HRs of 0.91 (95% CI, 0.85-0.98) for those aged 18–64 years and 0.82 (95% CI, 0.77-0.88) for those ≥65 years. The effect was observed across different BMI categories, with HRs of 0.77 (95% CI, 0.67-0.88) for BMI <30 kg/m² and 0.90 (95% CI, 0.82-0.99) for BMI ≥30 kg/m². Significant effects were observed in patients with and without liver cirrhosis (HR, 0.84,; 95% CI, 0.81-0.89 and HR, 0.84; 95% CI, 0.80-0.89 respectively), those without NASH (HR, 0.83; 95% CI, 0.79-0.87), and in the presence or absence of heart failure (HR, 0.78; 95% CI, 0.71-0.84 and HR, 0.83; 95% CI, 0.78-0.89 respectively), in patients with and without T2DM (HR, 0.84; 95% CI, 0.79-0.89 and HR, 0.84; 95% CI, 0.76-0.93 respectively) and in those without CKD (HR, 0.82; 95% CI, 0.77-0.87) and with CKD (HR, 0.82; 95% CI, 0.74-0.90). The lower risk was observed for those with NASH, but the difference was non-significant (HR, 0.88; 95% CI, 0.72-1.06). **Figure 3** illustrates subgroup analysis of primary outcomes between empagliflozin and dapagliflozin groups.

The interaction analysis revealed no significant differences in treatment effects across most subgroups, including sex (p for interaction=0.14), liver cirrhosis (p for interaction=0.92), NASH (p for interaction=0.60), heart failure (p for interaction=0.17), T2DM (p for interaction=1.00), and CKD (p for interaction=0.95). However, significant interactions were observed for age groups (p for interaction=0.04), and there was a borderline significant interaction for BMI categories (p for interaction=0.06).

### Secondary outcomes

Compared to the dapagliflozin group, the empagliflozin group demonstrated significantly lower risks of all-cause hospitalization (HR, 0.84; 95% CI, 0.79–0.88), all-cause mortality (HR, 0.79; 95% CI, 0.66-0.96), MACE (HR, 0.88; 95% CI, 0.78–0.99), and MAKE (HR, 0.63; 95% CI, 0.47–0.86) (**Table 2**). However, there was no significant difference in the risk of decompensated hepatic events between groups (HR, 1.01; 95% CI, 0.81–1.27).

### Negative control

Our study revealed no significant differences in the hazard of negative control outcomes between the empagliflozin and dapagliflozin groups for skin cancer, sensorineural hearing loss, and lumbar radiculopathy (**eTable 4**).

## Discussion

This retrospective study of 26,548 patients with metabolic-associated steatotic liver disease (MASLD) assessed the effects of two SGLT2 inhibitors - empagliflozin and dapagliflozin - on clinical outcomes. Compared to dapagliflozin, empagliflozin was associated with a 16% lower risk of composite outcomes, including all-cause hospitalization, all-cause mortality, MACE, MAKE, and decompensated hepatic events. These benefits of empagliflozin were consistently observed across most stratified analyses. Specifically, empagliflozin was linked to a 16% reduction in all-cause hospitalization, a 21% reduction in all-cause mortality, a 12% reduction in MACE, and a 37% reduction in MAKE. Despite previous studies (10–13) have highlighted the potential role of SGLT2 inhibitors for MASLD patients, this study is the first to directly compare the effects of different SGLT2 inhibitors. Our findings suggest that empagliflozin may offer additional benefits over dapagliflozin, potentially making it the preferred SGLT2 inhibitor option for MASLD treatment.

MASLD is a prevalent and progressive condition closely linked to metabolic comorbidities such as obesity, T2DM, and dyslipidemia (17). his disease poses significant clinical challenges due to its association with increased risks of cardiovascular complications, liver fibrosis progression, cirrhosis, and hepatocellular carcinoma (17–19). As a result, MASLD is associated with higher rates of morbidity and mortality, highlighting the urgent need for effective treatments to mitigate these risks (18–20). In this context, this large-scale study offers the first comparative analysis of clinical outcomes among different SGLT2 inhibitors in MASLD patients. Findings suggest that empagliflozin may be the preferred SGLT2 inhibitor option, though prospective randomized controlled trials are necessary to confirm these results. This study provides valuable guidance for clinicians in selecting SGLT2 inhibitor therapy for MASLD patients to optimize treatment outcomes.

Several studies (21–24) had compared the effect of empagliflozin versus dapagliflozin; however, the findings are not consistent. A Scandinavian cohort study including 141,065 new users of empagliflozin and 58,306 new users of dapagliflozin among patients with T2DM found that there were no significant differences between groups in terms of MACE (HR, 1.02; 95% CI, 0.97-1.08), heart failure (HR, 1.05; 95% CI, 0.97-1.14) and serious renal events (HR, 0.97; 95% CI, 0.87-1.07) between groups (21). In contrast, a prospective study assessed the long-term effectiveness and safety of empagliflozin and dapagliflozin in inadequately controlled T2DM showed that compared to dapagliflozin, empagliflozin was associated with significantly greater HbA1c and body weight reduction, and also had beneficial effects on HDL cholesterol and LDL cholesterol (all P < 0.05) (22). However, there were no significant differences in the overall incidence of adverse events, cardiovascular events and mortality between the two groups (22). In Greece, a health economic model was used to project the clinical outcome of patients with T2DM and established cardiovascular diseases, and demonstrated that empagliflozin was associated with longer mean survival (17.23 life years with empagliflozin vs 16.07 life years with dapagliflozin) and reduced rate of cardiovascular mortality resulting in 0.48 more quality-adjusted life years (9.27 vs 8.79) (23). In Taiwan, a multi-institutional cohort study including 12,681 new users of SGLT2i for T2DM showed that compared to empagliflozin users, dapagliflozin exhibited similar risks for primary composite outcome of cardiovascular death, myocardial infarction, ischemic stroke and heart failure (adjusted HR, 0.91; 95% CI, 0.73-1.14) (24). Regarding individual outcomes, no differences were found between groups in cardiovascular death (adjusted HR, 0.54; 95%, CI 0.14-2.12), myocardial infarction (adjusted HR, 0.77; 95% CI, 0.49-1.19) and ischemic stroke (adjusted HR, 1.15; 95% CI, 0.80-1.65), but a lower risk of heart failure was observed for dapagliflozin users (adjusted HR, 0.68; 95% CI, 0.49-0.95) (24). Critically, our study distinguishes itself from previous research by focusing specifically on patients with MASLD, whereas prior comparisons predominantly centered on patients with T2DM. This nuanced approach provides novel insights into the differential effects of SGLT2is in a metabolic liver disease context. While existing studies (21–24) have yielded conflicting results in diabetic populations, our research offers a more targeted analysis of these medications’ impacts on liver-related and systemic outcomes in MASLD patients. The consistent superiority of empagliflozin across multiple outcome measures suggests a potentially more beneficial therapeutic profile for this specific patient population, warranting further investigation and potential clinical implementation.

The underlying mechanisms for the observed differences between empagliflozin and dapagliflozin remain to be fully elucidated. Although both drugs share the same pharmacologic class, they differ in pharmacokinetic properties and selectivity for SGLT2i. Empagliflozin has greater SGLT2 selectivity, which may translate into more favorable renal and cardiovascular effects (25, 26). In addition, variations in tissue distribution, metabolic effects, and pleiotropic actions, such as modulation of oxidative stress, inflammation, and endothelial function (27–29), may contribute to the superior outcomes observed with empagliflozin in this MASLD population. Nevertheless, these hypotheses require further mechanistic and translational studies to confirm.

This study has several notable strengths. First, it utilized a large and diverse cohort from the TriNetX network, including 26,548 patients, which enhances the generalizability of the findings across various demographic groups. By applying 1:1 PSM, the study effectively minimized potential confounding biases, allowing for a more precise comparison between empagliflozin and dapagliflozin in MASLD patients. Additionally, while most previous studies focused primarily on T2DM populations, this research specifically targets patients with MASLD, addressing a significant gap in understanding the differential effects of SGLT2is on liver health in this population. Moreover, the study’s comprehensive assessment of both primary composite outcomes and a range of secondary outcomes, including all-cause hospitalization, mortality, MACE and MAKE, offers a holistic view of empagliflozin’s clinical advantages over dapagliflozin.

Despite these strengths, the study has certain limitations. First, the retrospective design introduces potential selection and information biases that could influence the results, even with the use of PSM. Second, the absence of liver-specific biomarkers for hepatic steatosis, inflammation, and fibrosis, which restricts the ability to directly assess improvements in liver health. Third, while the findings are significant, the study’s relatively short follow-up period limits conclusions regarding the long-term efficacy and safety of empagliflozin compared to dapagliflozin in patients with MASLD. Forth, although rigorous adjustment methods were applied, the possibility of residual confounding due to unmeasured variables, such as dietary habits and physical activity, remains, as these factors are not captured in the TriNetX database. Fifth, among patients receiving empagliflozin, most were prescribed the 10 mg dose in this study. In contrast, only a small number received the 25 mg dose, likely due to our new-user study design. Patients on 25 mg may have initially started on 10 mg and were later titrated upward because of inadequate glycemic control. Accordingly, the subgroup receiving 10 mg showed results consistent with the primary analysis, demonstrating a lower risk of the primary composite outcome (HR, 0.87; 95% CI, 0.84–0.90). However, no significant association was observed for those receiving 25 mg, likely due to the small sample size. Lastly, to ensure a direct head-to-head comparison, we restricted the cohort to new users of empagliflozin or dapagliflozin and excluded patients with prior use of other SGLT2i. Consequently, a pooled SGLT2 inhibitor comparator group was not included. This design minimized potential confounding but the absence of a non–SGLT2 inhibitor comparator group limits broader contextual interpretation. Future studies are warranted to compare these agents with other oral antidiabetic drugs in the MASLD population.

## Conclusion

In conclusion, this study provides valuable evidence in favor of empagliflozin as a preferred option over dapagliflozin for managing MASLD in patients with concurrent diabetes. Empagliflozin was associated with a reduced risk of the primary composite outcome as well as almost of all secondary outcomes, such as all-cause hospitalization and mortality, suggesting a potentially more beneficial therapeutic profile. These findings highlight empagliflozin’s promise as an effective treatment option for MASLD patients. However, to substantiate these results and establish a clearer role for empagliflozin in improving clinical outcomes in MASLD, future prospective studies with extended follow-up periods are necessary.

## Data Availability

The original contributions presented in the study are included in the article/**Supplementary Material**. Further inquiries can be directed to the corresponding authors.
