# Supplementary material for: Comparative effectiveness of empagliflozin versus dapagliflozin in adults with metabolic dysfunction-associated steatotic liver disease
Source: Front Endocrinol (Lausanne). 2025 Oct 13;16:1669613. doi: 10.3389/fendo.2025.1669613 (PMC12554547; doi:10.3389/fendo.2025.1669613)
Supplement: Supplementary file 1 [file Table1.docx]

Supplemental Online Content

**eTable 1.** Demographic, Diagnostic, Procedural, Medication, Visit, and Laboratory Codes Used in the Definition of the Cohorts

**eTable 2.** Demographic, Diagnostic, and Laboratory Codes Used in the Definition of Covariates

**eTable 3.** Diagnostic, Visit, and Procedural Codes Used in the Definition of Outcomes

**eTable 4.** Results for negative control outcomes and analysis with negative control exposure

**eTable 1.** Demographic, Diagnostic, Procedural, Medication, Visit, and Laboratory Codes Used in the Definition of the Cohorts

| **Category** | **Code** | **Description** |
| --- | --- | --- |
| **dapagliflozin group** | | |
| **#1**: At least 18 years old | | |
| Demographics | Age | Age (at least 18 years) |
| **#2**: Patients with metabolic dysfunction-associated steatotic liver disease (MASLD) treated with dapagliflozin but not other competitor drug including empagliflozin, linagliptin, ertugliflozin and canagliflozin. (# 2.2 must be fulfilled within 6 months before or any time after #2.1) | | |
| **#2.1**: Patients with metabolic dysfunction-associated steatotic liver disease (MASLD) treated with dapagliflozin. (must fit the both of the following section including diagnosis and medication) Date constraint: The terms in this group occurred between Jan 01, 2013 and Sep 30, 2024 | | |
| Diagnosis | MASLD | [(1) OR (2)] AND  [(3) OR (4) OR (5) OR (6) OR (7) OR (8) OR (9) OR (10) OR (11) OR (12) OR (13) OR (14) OR (15) OR (16)] |
|  | 1. UMLS: ICD-10-CM k76.0 | Fatty (change of) liver, not elsewhere classified |
|  | 1. UMLS: ICD-10-CM k75.01 | Nonalcoholic steatohepatitis (NASH) |
|  | 1. LOINC 39156-5 Body Mass Index \| | ≥ 25 kg/m^2^ |
|  | 1. LOINC 8280-0 Waist Circumference at umbilicus by Tape measure | ≥ 94 cm |
|  | 1. LOINC 56115-9 Waist Circumference by NCFS | ≥ 94 cm |
|  | 1. TNX Curated 9037 Hemoglobin Alc/ Hemoglobin. total in Blood | ≥ 5.7% |
|  | 1. IcD10cM E11 | Type 2 diabetes mellitus |
|  | 1. NLM:ATC A10A | insulins and analogues |
|  | 1. NLM:ATC A10B | blood glucose lowering drugs, excluding insulins |
|  | 1. LOINC 1558-6 | Fasting glucose [Mass/volume] in Serum or Plasma ≥ 100 mg/dL |
|  | 1. TNX Curated 9085 Blood Pressure, Systolic | ≥130 mm[Hg] |
|  | 1. TNX Curated 9086 Blood Pressure, Diastolic | ≥85mmlHg \| |
|  | 1. NLM:ATC C82 | antihypertensives |
|  | 1. TNX Curated 9004 Triglyceride [Mass/volume] in Serum, Plasma or Blood | ≥ 150 mg/dL |
|  | 1. NLM:ATC C18 | lipid modifying agents |
|  | 1. TNX Curated 9001 Cholesterol in HDL [Mass/volume] in Serum or Plasma | ≥ 40mg/dL |
| Medication | NLM:RXNORM:1488564 | dapagliflozin |
| **#2.2**: Not receiving empagliflozin, linagliptin, ertugliflozin and canagliflozin (cannot have the following) | | |
| medication | NLM:RXNORM:1545653 | empagliflozin |
|  | NLM:RXNORM:1100699 | linagliptin |
|  | NLM:RXNORM:1992672 | ertugliflozin |
|  | NLM:RXNORM:1373458 | canagliflozin |
| **#3**: Without recent major adverse cardiovascular events (cannot have any of the following) #3 must be fulfilled within 60 days on or before #2.1 | | |
| Diagnosis | UMLS:ICD10CM:I21 | Acute myocardial infarction |
| Diagnosis | UMLS:ICD10CM:I22 | Subsequent ST elevation (STEMI) and non-ST elevation (NSTEMI) myocardial infarction |
| Diagnosis | UMLS:ICD10CM:I61 | Nontraumatic intracerebral hemorrhage |
| Diagnosis | UMLS:ICD10CM:I62 | Other and unspecified nontraumatic intracranial hemorrhage |
| Diagnosis | UMLS:ICD10CM:I63 | Cerebral infarction |
| **#4**: Without chronic liver events or Type 1 diabetes mellitus (cannot have any of the following) #4 must be fulfilled within 60 days on or before #2.1 | | |
| Diagnosis | UMLS: ICD-10-CM K70 | Alcoholic liver disease |
| Diagnosis | UMLS: ICD-10-CM K73 | Chronic hepatitis, not elsewhere classified |
| Diagnosis | UMLS: ICD-10-CM K75.4 | Autoimmune hepatitis |
| Diagnosis | UMLS: ICD-10-CM B15-B19 | Viral hepatitis |
| Diagnosis | UMLS: ICD-10-CM K71 | Toxic liver disease |
| Diagnosis | UMLS: ICD-10-CM K75.0 | Abscess of liver |
| Diagnosis | UMLS: ICD-10-CM K75.2 | Nonspecific reactive hepatitis |
| Diagnosis | UMLS: ICD-10-CM K75.3 | Granulomatous hepatitis, not elsewhere classified |
| Diagnosis | UMLS: ICD-10-CM PCS HZ2 | Detoxification Services |
| Diagnosis | UMLS: ICD-10-CM I82.0 | Budd-Chiari syndrome |
| Diagnosis | UMLS: ICD-10-CM K83.01 | Primary sclerosing cholangitis |
| Diagnosis | UMLS: ICD-10-CM E83.01 | Wilson's disease |
| Diagnosis | UMLS: ICD-10-CM E10 | Type 1 diabetes mellitus |
| **#5**: Without end stage renal disease or decompensated liver cirrhosis (cannot have any of the following) #4 must be fulfilled within 60 days on or before #2.1 | | |
|  | ICD-10-CM N18.5  R \| | Chronic kidney disease, stage 5 |
|  | TNX Curated 8001 Glomerular  Filtration rate/1.73 sq M.predicted  [Volume Rate/Area] in Serum, Plasma or Blood by Creatinine-  based formula(MDRD) | ≤ 14.99 mL/min/{1.73_m2} |
|  | ICD-10-CM N18.6 | End stage renal disease |
|  | ICD-10-CM I85.01 | Esophageal varices with bleeding |
|  | ICD-10-CM I86.4 | Gastric varices |
|  | ICD-10-CM k76.82 Hepatic | encephalopathy |
|  | ICD-10-CM k72 | Hepatic failure, not elsewhere classified |
|  | ICD-10-CM K65.2 | Spontaneous bacterial peritonitis |
|  | ICD-10-CM R18 | Ascites |
|  | ICD-10-CM K76.7 | Hepatorenal syndrome |
| #6: Incident user (cannot have the following) #6 must be fulfilled before #2.1 | | |
| Medication | NLM:RXNORM:1488564 | dapagliflozin |
| **Empagliflozin group** | | |
| **#1**: At least 18 years old | | |
| Demographics | Age | Age (at least 18 years) |
| **#2**: Patients with metabolic dysfunction-associated steatotic liver disease (MASLD) treated with dapagliflozin but not other competitor drug including dapagliflozin, linagliptin, ertugliflozin and canagliflozin. (# 2.2 must be fulfilled within 6 months before or any time after #2.1) | | |
| **#2.1**: Patients with metabolic dysfunction-associated steatotic liver disease (MASLD) treated with dapagliflozin. (must fit the both of the following section including diagnosis and medication) Date constraint: The terms in this group occurred between Jan 01, 2013 and Sep 30, 2024 | | |
| Diagnosis | MASLD | [(1) OR (2)] AND  [(3) OR (4) OR (5) OR (6) OR (7) OR (8) OR (9) OR (10) OR (11) OR (12) OR (13) OR (14) OR (15) OR (16)] |
|  | 1. UMLS: ICD-10-CM k76.0 | Fatty (change of) liver, not elsewhere classified |
|  | 1. UMLS: ICD-10-CM k75.01 | Nonalcoholic steatohepatitis (NASH) |
|  | 1. LOINC 39156-5 Body Mass Index \| | ≥ 25 kg/m^2^ |
|  | 1. LOINC 8280-0 Waist Circumference at umbilicus by Tape measure | ≥ 94 cm |
|  | 1. LOINC 56115-9 Waist Circumference by NCFS | ≥ 94 cm |
|  | 1. TNX Curated 9037 Hemoglobin Alc/ Hemoglobin. total in Blood | ≥ 5.7% |
|  | 1. IcD10cM E11 | Type 2 diabetes mellitus |
|  | 1. NLM:ATC A10A | insulins and analogues |
|  | 1. NLM:ATC A10B | blood glucose lowering drugs, excluding insulins |
|  | 1. LOINC 1558-6 | Fasting glucose [Mass/volume] in Serum or Plasma ≥ 100 mg/dL |
|  | 1. TNX Curated 9085 Blood Pressure, Systolic | ≥130 mm[Hg] |
|  | 1. TNX Curated 9086 Blood Pressure, Diastolic | ≥85mmlHg \| |
|  | 1. NLM:ATC C82 | ≥ antihypertensives |
|  | 1. TNX Curated 9004 Triglyceride [Mass/volume] in Serum, Plasma or Blood | ≥ 150 mg/dL \| |
|  | 1. NLM:ATC C18 | lipid modifying agents |
|  | 1. TNX Curated 9001 Cholesterol in HDL [Mass/volume] in Serum or Plasma | ≥ 40mg/dL |
| Medication | NLM:RXNORM:1545653 | empagliflozin |
| **#2.2**: Not receiving empagliflozin, linagliptin, ertugliflozin and canagliflozin (cannot have the following) | | |
| Medication | NLM:RXNORM:1488564 | dapagliflozin |
| Medication | NLM:RXNORM:1100699 | linagliptin |
| Medication | NLM:RXNORM:1992672 | ertugliflozin |
| Medication | NLM:RXNORM:1373458 | canagliflozin |
| **#3**: Without recent major adverse cardiovascular events (cannot have any of the following) #3 must be fulfilled within 60 days on or before #2.1 | | |
| Diagnosis | UMLS:ICD10CM:I21 | Acute myocardial infarction |
| Diagnosis | UMLS:ICD10CM:I22 | Subsequent ST elevation (STEMI) and non-ST elevation (NSTEMI) myocardial infarction |
| Diagnosis | UMLS:ICD10CM:I61 | Nontraumatic intracerebral hemorrhage |
| Diagnosis | UMLS:ICD10CM:I62 | Other and unspecified nontraumatic intracranial hemorrhage |
| Diagnosis | UMLS:ICD10CM:I63 | Cerebral infarction |
| **#4**: Without chronic liver events or Type 1 diabetes mellitus (cannot have any of the following) #4 must be fulfilled within 60 days on or before #2.1 | | |
| Diagnosis | UMLS: ICD-10-CM K70 | Alcoholic liver disease |
| Diagnosis | UMLS: ICD-10-CM K73 | Chronic hepatitis, not elsewhere classified |
| Diagnosis | UMLS: ICD-10-CM K75.4 | Autoimmune hepatitis |
| Diagnosis | UMLS: ICD-10-CM B15-B19 | Viral hepatitis |
| Diagnosis | UMLS: ICD-10-CM K71 | Toxic liver disease |
| Diagnosis | UMLS: ICD-10-CM K75.0 | Abscess of liver |
| Diagnosis | UMLS: ICD-10-CM K75.2 | Nonspecific reactive hepatitis |
| Diagnosis | UMLS: ICD-10-CM K75.3 | Granulomatous hepatitis, not elsewhere classified |
| Diagnosis | UMLS: ICD-10-CM PCS HZ2 | Detoxification Services |
| Diagnosis | UMLS: ICD-10-CM I82.0 | Budd-Chiari syndrome |
| Diagnosis | UMLS: ICD-10-CM K83.01 | Primary sclerosing cholangitis |
| Diagnosis | UMLS: ICD-10-CM E83.01 | Wilson's disease |
| Diagnosis | UMLS: ICD-10-CM E10 | Type 1 diabetes mellitus |
| **#5**: Without end stage renal disease or decompensated liver cirrhosis (cannot have any of the following) #4 must be fulfilled within 60 days on or before #2.1 | | |
| Diagnosis | ICD-10-CM N18.5 | Chronic kidney disease, stage 5 |
| Laboratory | TNX Curated 8001 Glomerular  Filtration rate/1.73 sq M.predicted  [Volume Rate/Area] in Serum, Plasma or Blood by Creatinine-  based formula(MDRD) | ≤ 14.99 mL/min/{1.73_m2} |
| Diagnosis | ICD-10-CM N18.6 | End stage renal disease |
| Diagnosis | ICD-10-CM I85.01 | Esophageal varices with bleeding |
| Diagnosis | ICD-10-CM I86.4 | Gastric varices |
| Diagnosis | ICD-10-CM k76.82 Hepatic | encephalopathy |
| Diagnosis | ICD-10-CM k72 | Hepatic failure, not elsewhere classified |
| Diagnosis | ICD-10-CM K65.2 | Spontaneous bacterial peritonitis |
| Diagnosis | ICD-10-CM R18 | Ascites |
| Diagnosis | ICD-10-CM K76.7 | Hepatorenal syndrome |
| #6: Incident user (cannot have the following) #6 must be fulfilled before #2.1 | | |
| Medication | NLM:RXNORM:1545653 | empagliflozin |

**eTable 2.** Demographic, Diagnostic, and Laboratory Codes Used in the Definition of Covariates

| **Category** | **Code** | **Description** |
| --- | --- | --- |
| Demographics | AI | Age at index |
| Demographics | F | Female |
| Demographics | 2106-3 | White |
| Demographics | UNK | Unknown Race |
| Demographics | 2054-5 | Black or African American |
| Demographics | 2028-9 | Asian |
| Demographics | 2131-1 | Other Race |
| Diagnosis | I10-I16 | Hypertensive diseases |
| Diagnosis | I20-I25 | Ischemic heart diseases |
| Diagnosis | I60-I69 | Cerebrovascular diseases |
| Diagnosis | I50 | Heart failure |
| Diagnosis | I48 | Atrial fibrillation and flutter |
| Diagnosis | I73.9 | Peripheral vascular disease, unspecified |
| Diagnosis | E78 | Disorders of lipoprotein metabolism and other lipidemias |
| Diagnosis | I10 | Essential (primary) hypertension |
| Diagnosis | E66 | Overweight and obesity |
| Diagnosis | F17 | Nicotine dependence |
| Diagnosis | F10 | Alcohol related disorders |
| Diagnosis | J40-J4A | Chronic lower respiratory diseases |
| Diagnosis | N18.6 | End stage renal disease |
| Diagnosis | C00-D49 | Neoplasms |
| Diagnosis | M30-M36 | Systemic connective tissue disorders |
| Diagnosis | K74.6 | Other and unspecified cirrhosis of liver |
| Diagnosis | K74.0 | Hepatic fibrosis |
| Diagnosis | K75.81 | Nonalcoholic steatohepatitis (NASH) |
| Diagnosis | K76.0 | Fatty (change of) liver, not elsewhere classified |
| Diagnosis | E11 | Type 2 diabetes mellitus |
| Medication | C03 | Diuretics |
| Medication | C07 | Beta blocking agents |
| Medication | C08 | Calcium channel blockers |
| Medication | C02 | Antihypertensives |
| Medication | C09 | Agents acting on the renin-angiotensin system |
| Medication | C10AA | Hmg coa reductase inhibitors |
| Medication | C01B | Antiarrhythmics, class i and iii |
| Medication | A10A | Insulins and analogues |
| Medication | A10BA | Biguanides |
| Medication | A10BB | Sulfonylureas |
| Medication | A10BH | Dipeptidyl peptidase 4 (dpp-4) inhibitors |
| Medication | A10BG | Thiazolidinediones |
| Medication | A10BJ | Glucagon-like peptide-1 (glp-1) analogues |
| Laboratory | 8001 | Glomerular filtration rate by creatinine-based formula |
| Laboratory | 9037 | Hemoglobin A1c/hemoglobin in blood |

**eTable 3.** Diagnostic, Visit, and Procedural Codes Used in the Definition of Outcomes

| **Category** | **Code** | **Description** |
| --- | --- | --- |
| **#1**: decompensated liver cirrhosis (have any of the following) | | |
| Diagnosis | UMLS:ICD10CM:I85.01 | Esophageal varices with bleeding |
| Diagnosis | UMLS:ICD10CM:I86.4 | Gastric varices |
| Diagnosis | UMLS:ICD10CM:K76.82 | Hepatic encephalopathy |
| Diagnosis | UMLS:ICD10CM:K72 | Hepatic failure, not elsewhere classified |
| Diagnosis | UMLS:ICD10CM:R18 | Ascites |
| Diagnosis | UMLS:ICD10CM:K65.2 | Spontaneous bacterial peritonitis |
| Diagnosis | UMLS:ICD10CM:K76.7 | Hepatorenal syndrome |
| **#2**: All cause mortality (have any of the following) | | |
| Demographics | Deceased | Deceased |
| Diagnosis | UMLS:ICD10CM:R99 | Ill-defined and unknown cause of mortality |
| **#3**: Major adverse cardiovascular event (have any of the following) | | |
| Diagnosis | UMLS:ICD10CM:I21 | Acute myocardial infarction |
| Diagnosis | UMLS:ICD10CM:I22 | Subsequent ST elevation (STEMI) and non-ST elevation (NSTEMI) myocardial infarction |
| Diagnosis | UMLS:ICD10CM:I61 | Nontraumatic intracerebral hemorrhage |
| Diagnosis | UMLS:ICD10CM:I62 | Other and unspecified nontraumatic intracranial hemorrhage |
| Diagnosis | UMLS:ICD10CM:I63 | Cerebral infarction |
| Diagnosis | UMLS:ICD10CM:I46.2 | Cardiac arrest due to underlying cardiac condition |
| Diagnosis | UMLS:ICD10CM:I46.9 | Cardiac arrest, cause unspecified |
| **#4**: Major adverse kidney event (have any of the following) | | |
| Diagnosis | UMLS:ICD10CM:N18.5 | Chronic kidney disease, stage 5 |
| Diagnosis | UMLS:ICD10CM:N18.6 | End stage renal disease |
| Diagnosis | UMLS:ICD10CM:N19 | Unspecified kidney failure |
| Diagnosis | UMLS:ICD10CM:Z99.2 | Dependence on renal dialysis |
| Procedure | UMLS:CPT:1012740 | Dialysis Services and Procedures |
| Procedure | UMLS:CPT:1029674 | Dialysis Circuit Procedures |
| **#5**: Composite outcome (have any of the following) | | |
| Diagnosis | UMLS:ICD10CM:I85.01 | Esophageal varices with bleeding |
| Diagnosis | UMLS:ICD10CM:I86.4 | Gastric varices |
| Diagnosis | UMLS:ICD10CM:K76.82 | Hepatic encephalopathy |
| Diagnosis | UMLS:ICD10CM:K72 | Hepatic failure, not elsewhere classified |
| Diagnosis | UMLS:ICD10CM:R18 | Ascites |
| Diagnosis | UMLS:ICD10CM:K65.2 | Spontaneous bacterial peritonitis |
| Diagnosis | UMLS:ICD10CM:K76.7 | Hepatorenal syndrome |
| Diagnosis | UMLS:ICD10CM:I21 | Acute myocardial infarction |
| Diagnosis | UMLS:ICD10CM:I22 | Subsequent ST elevation (STEMI) and non-ST elevation (NSTEMI) myocardial infarction |
| Diagnosis | UMLS:ICD10CM:I61 | Nontraumatic intracerebral hemorrhage |
| Diagnosis | UMLS:ICD10CM:I62 | Other and unspecified nontraumatic intracranial hemorrhage |
| Diagnosis | UMLS:ICD10CM:I63 | Cerebral infarction |
| Diagnosis | UMLS:ICD10CM:I46.2 | Cardiac arrest due to underlying cardiac condition |
| Diagnosis | UMLS:ICD10CM:I46.9 | Cardiac arrest, cause unspecified |
| Diagnosis | UMLS:ICD10CM:N18.5 | Chronic kidney disease, stage 5 |
| Diagnosis | UMLS:ICD10CM:N18.6 | End stage renal disease |
| Diagnosis | UMLS:ICD10CM:N19 | Unspecified kidney failure |
| Diagnosis | UMLS:ICD10CM:Z99.2 | Dependence on renal dialysis |
| Procedure | UMLS:CPT:1012740 | Dialysis Services and Procedures |
| Procedure | UMLS:CPT:1029674 | Dialysis Circuit Procedures |
| Demographics | Deceased | Deceased |
| Diagnosis | UMLS:ICD10CM:R99 | Ill-defined and unknown cause of mortality |
| Visit | UMLS:HL7V3.0:VisitType:SS | Visit: Short Stay |
| Visit | UMLS:HL7V3.0:VisitType:IMP | Visit: Inpatient Encounter |
| Visit | UMLS:HL7V3.0:VisitType:NONAC | Visit: Inpatient Non-acute |
| Visit | UMLS:HL7V3.0:VisitType:ACUTE | Visit: Inpatient Acute |
| **#6**: hospitalization (have any of the following) | | |
| Visit | UMLS:HL7V3.0:VisitType:SS | Visit: Short Stay |
| Visit | UMLS:HL7V3.0:VisitType:IMP | Visit: Inpatient Encounter |
| Visit | UMLS:HL7V3.0:VisitType:NONAC | Visit: Inpatient Non-acute |
| Visit | UMLS:HL7V3.0:VisitType:ACUTE | Visit: Inpatient Acute |
| **#7**: negative outcome, skin cancer (have any of the following) | | |
| Diagnosis | UMLS:ICD10CM:C44 | Other and unspecified malignant neoplasm of skin |
| **#8**: negative outcome, sensorineural hearing loss (have any of the following) | | |
| Diagnosis | UMLS:ICD10CM:H90 | Conductive and sensorineural hearing loss |
| **#9**: negative outcome, lumbar radiculopathy (have any of the following) | | |
| Diagnosis | UMLS:ICD10CM:H54.16 | Radiculopathy, lumbar region |

**eTable 4.** Results for negative control outcomes and analysis with negative control exposure

| Outcome | No. of patients with outcome | | HR (95% CI) | *P* value |
| --- | --- | --- | --- | --- |
|  | Empagliflozin  (n = 12 951) | Dapagliflozin  (n = 12 951) |  |  |
| Skin cancer | 43 | 41 | 1.04 (0.68,1.59) | 0.861 |
| Sensorineural hearing loss | 123 | 114 | 1.08 (0.84,1.39) | 0.561 |
| Lumbar radiculopathy | 190 | 172 | 1.1 (0.9,1.35) | 0.365 |
